# Cancer Neoantigens: Challenges and Future Directions for Prediction, Prioritization, and Validation

**DOI:** 10.3389/fonc.2022.836821

**Published:** 2022-03-03

**Authors:** Elizabeth S. Borden, Kenneth H. Buetow, Melissa A. Wilson, Karen Taraszka Hastings

**Affiliations:** ^1^Department of Basic Medical Sciences, College of Medicine-Phoenix, University of Arizona, Phoenix, AZ, United States; ^2^Department of Research and Internal Medicine (Dermatology), Phoenix Veterans Affairs Health Care System, Phoenix, AZ, United States; ^3^School of Life Sciences, Arizona State University, Tempe, AZ, United States; ^4^Center for Evolution and Medicine, Arizona State University, Tempe, AZ, United States

**Keywords:** neoantigens (neoAgs), MHC class I, MHC class II, neoantigen prioritization, neoantigen prediction

## Abstract

Prioritization of immunogenic neoantigens is key to enhancing cancer immunotherapy through the development of personalized vaccines, adoptive T cell therapy, and the prediction of response to immune checkpoint inhibition. Neoantigens are tumor-specific proteins that allow the immune system to recognize and destroy a tumor. Cancer immunotherapies, such as personalized cancer vaccines, adoptive T cell therapy, and immune checkpoint inhibition, rely on an understanding of the patient-specific neoantigen profile in order to guide personalized therapeutic strategies. Genomic approaches to predicting and prioritizing immunogenic neoantigens are rapidly expanding, raising new opportunities to advance these tools and enhance their clinical relevance. Predicting neoantigens requires acquisition of high-quality samples and sequencing data, followed by variant calling and variant annotation. Subsequently, prioritizing which of these neoantigens may elicit a tumor-specific immune response requires application and integration of tools to predict the expression, processing, binding, and recognition potentials of the neoantigen. Finally, improvement of the computational tools is held in constant tension with the availability of datasets with validated immunogenic neoantigens. The goal of this review article is to summarize the current knowledge and limitations in neoantigen prediction, prioritization, and validation and propose future directions that will improve personalized cancer treatment.

## 1 Introduction

Neoantigens are tumor-specific mutated peptides that are key targets of the anti-cancer immune response, because neoantigens are not subject to immune tolerance (non-reactivity to self) (1–4). Three classes of cancer therapies reliant on the neoantigen expression and presentation by MHC are personalized neoantigen vaccines, adoptive T cell therapy, and immune checkpoint inhibitors. Personalized neoantigen vaccines have gained momentum in recent years because of their early success (5–9). Several approaches to vaccination have been attempted to date, including direct exposure to neoantigens (6), neoantigen-encoding RNA vaccines (7), and neoantigen-loaded dendritic cell vaccines (5). Regardless of the vaccination strategy, all personalized neoantigen vaccines rely on accurate prediction of immunogenic neoantigens, neoantigens that are presented by MHC and elicit a T cell-mediated immune response.

Adoptive T cell therapy has also demonstrated promise as a targeted immunotherapy. Adoptive T cell therapy includes transfer of tumor-infiltrating lymphocytes and T cells genetically modified to express a T cell receptor (TCR) or chimeric antigen receptor. Early attempts at adoptive T cell therapy focused on introducing T cells specific for tumor associated antigens including MAGE-A3 in melanoma and carcinoembryonic antigen (CEA) in colorectal cancer (10, 11). However, the lack of tumor-specificity of these antigens led to significant off target effects and severe toxicity. There is, therefore, growing interest in the application of neoantigen-specific adoptive T cell therapy to enhance T cell mediated tumor-destruction while reducing off target effects (1, 12–15). As for personalized neoantigen vaccines, adoptive T cell therapy specific to neoantigens relies on accurate prediction of immunogenic neoantigens.

Tumor-specific neoantigens are also the target of cancer immunotherapy with immune checkpoint inhibitors (16, 17). Immune checkpoint inhibitors, including monoclonal antibodies against PD-1 and CTLA-4, block inhibitory signals to the T cells to increase T cell-mediated tumor destruction (18). Unfortunately, immune checkpoint inhibitors are only effective in a subset of patients and are associated with immune-related adverse events. Thus, there is interest in predicting which patients will respond to treatment with a single immune checkpoint inhibitor and which would benefit from combination therapy. Several recent studies have demonstrated that the predicted immunogenic neoantigens are more strongly associated with response to immune checkpoint inhibition than mutational burden (19–23). Accurately determining the association of neoantigen immunogenicity with response to immune checkpoint inhibition relies on accurate prioritization of immunogenic neoantigens.

**Figure 1:**
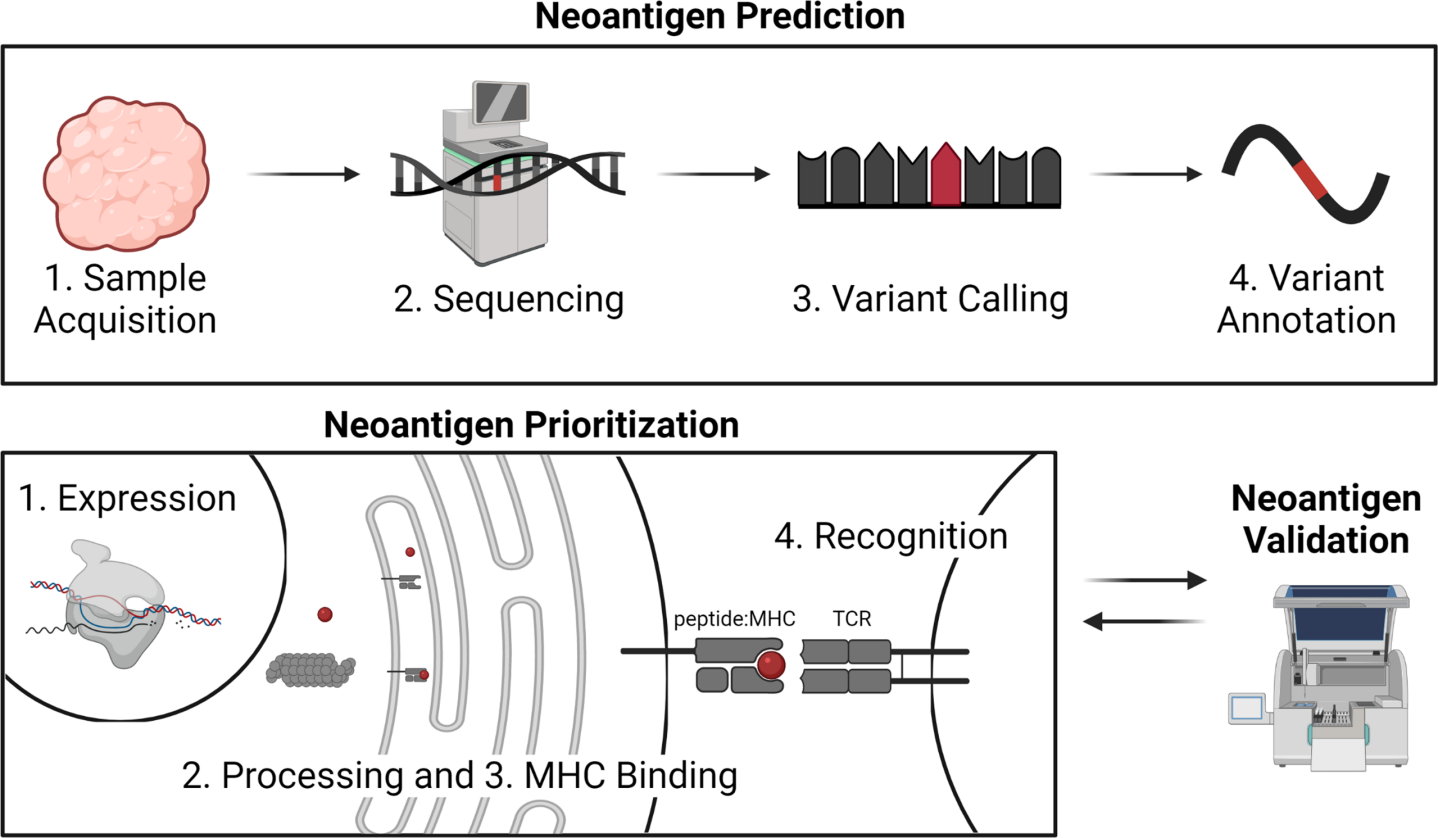
Overview of neoantigen prediction, prioritization, and validation. Neoantigen prediction relies on sample acquisition, high quality sequencing data, variant calling, and variant annotation. Neoantigen prioritization requires predicting some combination of the potential for the neoantigen to be expressed, processed, bound by MHC, and recognized by the T cell receptor (TCR). The development of neoantigen prioritization models relies on the availability of validated datasets of neoantigen immunogenicity. Figure created with BioRender.com.

Successful identification of immunogenic neoantigens using traditional genomic approaches requires a combination of neoantigen prediction and neoantigen prioritization (**Figure 1**). Neoantigen prediction requires sample acquisition, high quality sequencing data, prediction of the somatic mutations present in the tumor cell (variant calling), and accurate prediction of the neoantigens resulting from these somatic mutations (variant annotation). A few considerations for neoantigen identification include the tissue types to be sequenced, the best collection/preservation method for the tissues, and the type of sequencing data to be obtained. Additionally, one should decide on the types of mutations to be considered, appropriate methods by which to identify these mutations, and the most accurate annotation methods. Prioritization of immunogenic neoantigens relies on a thorough understanding of the characteristics of a neoantigen and the optimal ways of combining these characteristics to predict the potential of the neoantigen to elicit an immune response. For both MHC class I- and II-restricted neoantigens, characteristics that have been considered include expression of the neoantigen of interest, processing of the peptide including proteasomal cleavage and transport into the endoplasmic reticulum, binding of the neoantigen to MHC class I or II, and TCR recognition. Several tools are available for predicting each of these characteristics, and a variety of models have synthesized the characteristics into an overall immunogenicity score (21–26). We will review the available literature to guide decisions for each step in neoantigen prediction and prioritization and highlight areas for future research.

Datasets containing neoantigens that are validated to bind MHC class I or II and elicit a CD8+ or CD4+ T cell response are critical for assessing the overall performance of genomic pipelines and driving improved computational neoantigen identification. As the available datasets increase, models for neoantigen prioritization will be refined. Currently, many datasets are available for MHC class I-restricted neoantigens derived from single nucleotide variants (SNVs) and small insertions and deletions (indels). There are limited datasets available for MHC class II-restricted neoantigens and a lack of datasets for neoantigens derived from large indels, frameshifts, and gene fusions. We will summarize the available datasets and highlight ways to enhance future validation sets for continued improvement of neoantigen prediction and prioritization.

## 2 Neoantigen Prediction

### 2.1 Sample Acquisition

**Figure 2:**
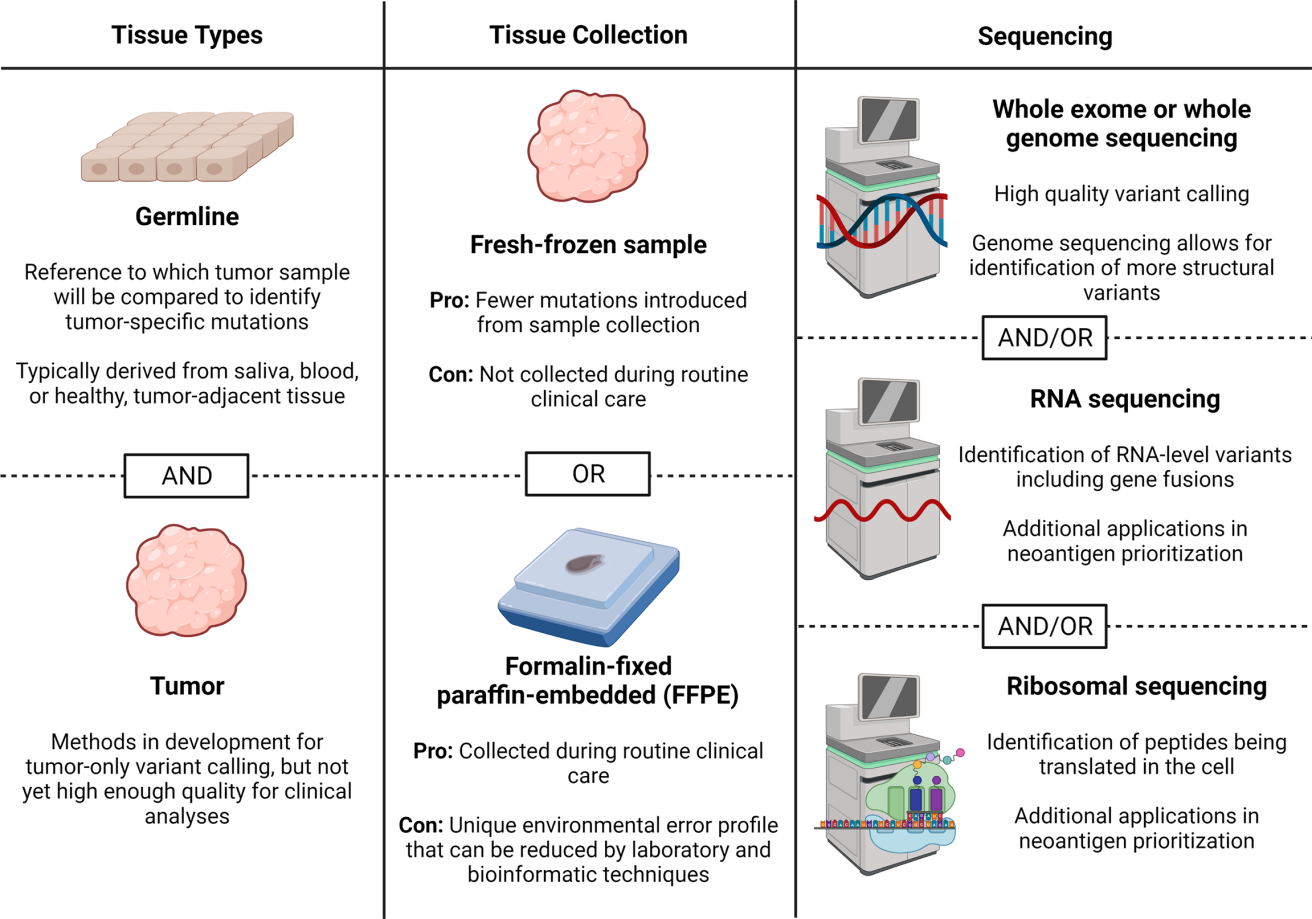
Sample collection and sequencing considerations. Here we describe considerations for obtaining sequencing data for neoantigen prediction including tissues needed, tissue collection method, and sequencing types. Figure created with BioRender.com.

Sample collection and sequencing are the first steps in performing neoantigen prediction and prioritization from DNA- or RNA-level mutations. While proteomic-based methods, which do not universally require the sequencing data presented here, have been created with direct profiling of peptides bound to MHC class I or II molecules, these are beyond the scope of this review article and have been discussed (27). Decisions related to sequencing can broadly be classified into the types of tissue needed, tissue collection methods, and types of sequencing. Here, we provide an up-to-date review of the literature to help guide each of these decisions (summarized in **Figure 2**).

The first consideration for sample acquisition is the tissues that need to be used to generate accurate somatic variant calls; specifically, whether a germline reference sample is required for variant calling. Typically, tumor and germline samples are compared to identify tumor-specific, somatic mutations. However, germline samples are not always available, especially for archived samples, though they can be collected in clinical settings. Therefore, there is continued interest in whether neoantigens can be identified in the absence of a germline sample. A patient-specific germline sample is currently the best available method to ensure that the variants being detected are due to true somatic mutations, rather than germline mutations (28). A few novel approaches have been suggested to reduce the number of germline variants identified without a germline reference sample (29–31). One such method uses tumor tissue only and assumptions about differences in the allelic frequency of the germline variants, compared to somatic variants to filter results. A germline heterozygous mutation should be closer to a 50% allelic frequency, whereas a somatic heterozygous mutation is likely to have less than a 50% allelic frequency because they won’t be present in germline tissue, only tumor samples. However, the assumption that a somatic heterozygous mutation will have less than a 50% allele frequency is complicated by copy number variations and stromal contamination, which are also accounted for in the model. Across seven test tumors, the sensitivity of the method ranged from 44-87%, which the authors acknowledge is too low to currently be applicable for clinical use (30). Another method performed variant calling for a tumor compared to 20 unmatched normal samples and kept variants that were identified in 90% of the comparisons. The variants were also filtered by 1) elimination of variants with the same allelic frequency as known germline mutations, 2) removal of variants from hard to map regions of the genome and 3) elimination of C→T and G→A mutations with low allelic frequencies. This method reported a 94% sensitivity, 99% specificity, and 76% positive predictive value (31). These numbers exceed those of other available tools and indicate that tumor-only variant calling may be an option for clinical applications in the future. However, these results were only validated for a set of stringently selected somatic mutations suggesting that further analysis would be needed to ensure that the results are stable for a more comprehensive set of tumor mutations. While the field of somatic variant calling is constantly improving, until the sensitivity and specificity of available methods improve, a germline sample is recommended.

When using a germline sample, a second question is which tissue source is the most accurate to use as the germline reference. Options that have been frequently employed in the literature include saliva, blood, or tumor-adjacent tissue, but the source of germline tissue can affect which variants are called as tumor-specific. Each tissue has its advantages and disadvantages. Saliva has the advantage of being a readily available, non-invasive method for obtaining a germline DNA reference. However, two recent studies using whole genome sequencing (WGS) on saliva samples demonstrated a risk for contamination from bacteria and food DNA that can influence the read mapping and variant calling (32, 33). When aggregated across four patients, saliva resulted in the identification of 776 unique coding variants compared to 157 from blood. Manual inspection of a sampling of the saliva-only variants demonstrated that most were attributable to bacterial contamination (32). The risk of bacterial contamination may be lessened in whole exome sequencing (WES) where hybridization methods are used to capture exons; however, an older study demonstrated bacterial contamination in WES data (34). To our knowledge, there are no studies that assess the impact of bacterial contamination on variant calling from WES data.

While slightly more invasive than saliva collection, blood still has the advantage of being minimally invasive. A study on optimizing cancer genomics experiments suggests that blood may be the best germline reference for solid tumors. Blood is a different tissue origin from most solid tumors and may have a lower risk for tumor-in-normal contamination than tumor-adjacent tissue (35). While the advantage of no tumor-in-normal contamination could be undermined by circulating tumor cell contamination, examination of ten cancer types from the Cancer Genome Atlas (TCGA) demonstrated no detection of tumor-in-normal contamination across the 304 blood samples tested (36). The tested blood samples were from patients with untreated primary tumors, so the risk of circulating tumor cells may be greater in advanced and metastatic disease. A recently developed tool, DeTiN has also been suggested as a means of removing the tumor-in-normal contamination (36). DeTiN demonstrated increased true positive variant detection with no significant change in the false positive rate (36).

With regards to tumor-adjacent tissue, a factor to consider is the potential for shared mutations between the tumor and tumor-adjacent tissue (37). One cause of these shared mutations could be exposure to a shared carcinogen. For example, recent work in skin cancer has demonstrated that there are early mutations in non-tumor sun-exposed skin due to exposure to ultraviolet radiation (38). Recent evidence has demonstrated the presence of somatic mutant clones within normal tissue (39–41). These somatic mutant clones can have numerous somatic mutations, a portion of which overlap with tumor mutations (41). It is unclear whether the presence of shared mutations within the tumor-adjacent sample will benefit or hinder the therapeutic utility of identified neoantigens. Shared mutations between tumor and tumor-adjacent tissue introduce the risk of eliminating neoantigens that occur in the cancer field and pre-cancerous lesions. However, the elimination of shared mutations may better facilitate tumor-specific targeting. Overall, if the goal is to maximize the number of tumor mutations identified, blood is the best available germline comparison for solid tumors, since it minimizes the risk of bacterial contamination and is the least likely to have a shared mutational profile. Additional research will be needed to assess the relative therapeutic benefits of neoantigens shared with the cancer field compared to unique tumor mutations.

Two common options for storing tissues used for neoantigen identification are fresh-frozen and formalin-fixed, paraffin-embedded (FFPE) samples, and each sample type has strengths and weaknesses. Fresh-frozen samples are attractive, because the samples have minimal processing that can affect DNA integrity; and typically, fresh-frozen samples can be used for both DNA and RNA isolation. However, fresh-frozen samples require a biobank setup to collect and are not part of routine clinical care. FFPE samples have the distinct advantage of being routinely collected in clinical settings, but have a characteristic set of mutations due to the preservation method and lack reliable RNA. A recent side-by-side comparison of variant calling in FFPE compared to fresh-frozen samples found that FFPE samples have ~5% more variants called than paired, fresh-frozen samples (42). The false discovery rate (FDR) was highly concentrated in the variants with low allelic frequency and is also predominated by C→T and G→A transitions due to deamination of methylated cytosine position (42), introduced by the FFPE process. Going forward, new approaches have been developed with DNA extraction kits that include enzymatic removal of cytosine deamination artifacts. Extraction with enzymatic removal of artifacts was shown to decrease the estimated FDR in low allelic frequency variants from 94.8% to 69.8% (42). Thus, fresh-frozen samples are preferred when possible; and when FFPE samples are used, DNA extraction protocols specific for FFPE samples are recommended.

Three possible bioinformatic methods can be applied to reduce false positives from FFPE damage: 1) taking the overlap from multiple variant callers, 2) eliminating low variant allele frequency variants, and 3) eliminating characteristic FFPE mutations. de Shaetzen *et al*. demonstrated increased agreement of variants called from FFPE with a set of high-confidence variants from fresh-frozen tumors when analyzing the overlap of at least two of the four variant callers employed: Strelka2, GATK Mutect2, Shimmer, and VarScan2 (43). A limitation to the consensus approach is that it may emphasize specificity over sensitivity and eliminate variants with potential clinical significance. Another approach (to be taken independently or in combination) is to filter out low allelic frequency calls, as one study suggested that the bulk of the false positives occur at lower frequency (42). However, a separate study demonstrated that highly reliable variants from fresh-frozen samples were generally represented at a lower allelic frequency in FFPE samples than in fresh-frozen samples (43). Additionally, the discrepancies between FFPE and fresh-frozen samples that remained after using the overlap of two out of four variant callers were demonstrated to be due to differences in the subclonal population (43). If low frequency variants are not errors, but rather represent subclonal populations, then eliminating the low frequency variants will result in a reduced ability to predict neoantigens. A newer method called Ideafix uses machine learning to consider a range of characteristics of the mutation to determine the likelihood of that mutation being an artifact of FFPE preservation. These characteristics include the variant allele frequency, the C→T mutational signature, the genomic context of the variant (based on flanking nucleotides that may increase the risk of deamination), and strand bias (whether the mutation is only identified on forward or reverse strand reads). Combining these features with a machine learning algorithm demonstrated an area under the receiver operator characteristics curve (AUC) of over 0.96 in two independent test datasets (44). Other recently developed models have taken a similar approach (45, 46). One potential challenge for these approaches is the inability to distinguish between true C→T mutations, including those enriched in ultraviolet light-induced tumors, and those that are artifacts due to FFPE processing. Overall, application of a newer model such as Ideafix may be helpful in eliminating FFPE artifacts while sacrificing minimal clinically relevant variants.

### 2.2 Sequencing

Each type of sequencing, including RNA sequencing (RNAseq), WGS, WES and combined approaches have potential advantages and limitations with regards to neoantigen prediction and prioritization. Traditionally, WGS or WES have been the preferred sequencing types for variant calling. WGS has the advantage of allowing for the identification of certain structural variants that are excluded by WES data (discussed below) but has the disadvantage of being more expensive than WES data (47). RNAseq data is a potential alternative to WGS or WES sequencing as it would allow for variant calling, as well as differential expression analysis and incorporation of mRNA expression data into neoantigen prioritization. Using RNAseq data for both variant calling and expression is a potentially attractive measure to reduce sequencing costs. However, methods for variant calling from RNAseq data have not been traditionally considered high enough quality to be used in isolation (48). Recent benchmarking demonstrated a low level of agreement between WES and RNAseq variants (49). One of the likely causes of the discrepancy between WES and RNAseq variants is that WES does not include all areas of the genome that may be transcribed, as another study demonstrated that ~71% of RNAseq-only variants occurred in regions not covered by the WES capture (48). Other possible causes include RNA-level modifications or differences in the read depth (49). To assess the performance of each method, variants called were compared to the COSMIC and dbSNP databases. The COSMIC database is a set of known cancer-specific mutations, whereas the dbSNP database is a database of variants known to exist in a healthy population. Therefore, enrichment of COSMIC-only variants reflects an increase in the likelihood of the mutation being a somatic mutation (49). Taking the intersection of RNAseq and WES variants led to enrichment of COSMIC-only variants, with 87.7% being COSMIC-only in the intersection approach compared to 39.5% in the WES and 3.0% in the RNAseq approach (49). A limitation acknowledged by the authors is that the COSMIC database is limited primarily to variants previously identified by WES analysis, so many RNAseq mutations may not be included in the COSMIC database. Overall, although WES-only approaches have been the most popular to date, there are possible advantages to RNAseq-based variant calling approaches. Further work is warranted to document the rates of true positive and false positive variant calls with RNAseq and WES approaches.

One final sequencing type to consider is a newer method for ribosomal profiling known as Ribo-seq, which allows for the specific transcription of all proteins being actively translated at the time of cell lysis (50). Ribo-seq has two potential advantages in the space of neoantigen prediction and prioritization. First, it has been proposed as a novel approach for detecting neoantigens derived from open reading frames by providing a snapshot of the reading frames of all proteins being translated in the cell (51). Secondly, Ribo-seq has the potential to give a more accurate expression profile for the purposes of neoantigen prioritization (discussed below). Given the novelty of the Ribo-seq approach, it does have the downside of being expensive and less readily available (51). Overall, further research is needed to fully explore the potential applications and advantages of Ribo-seq technology in neoantigen prioritization.

### 2.3 Variant Calling

**Figure 3:**
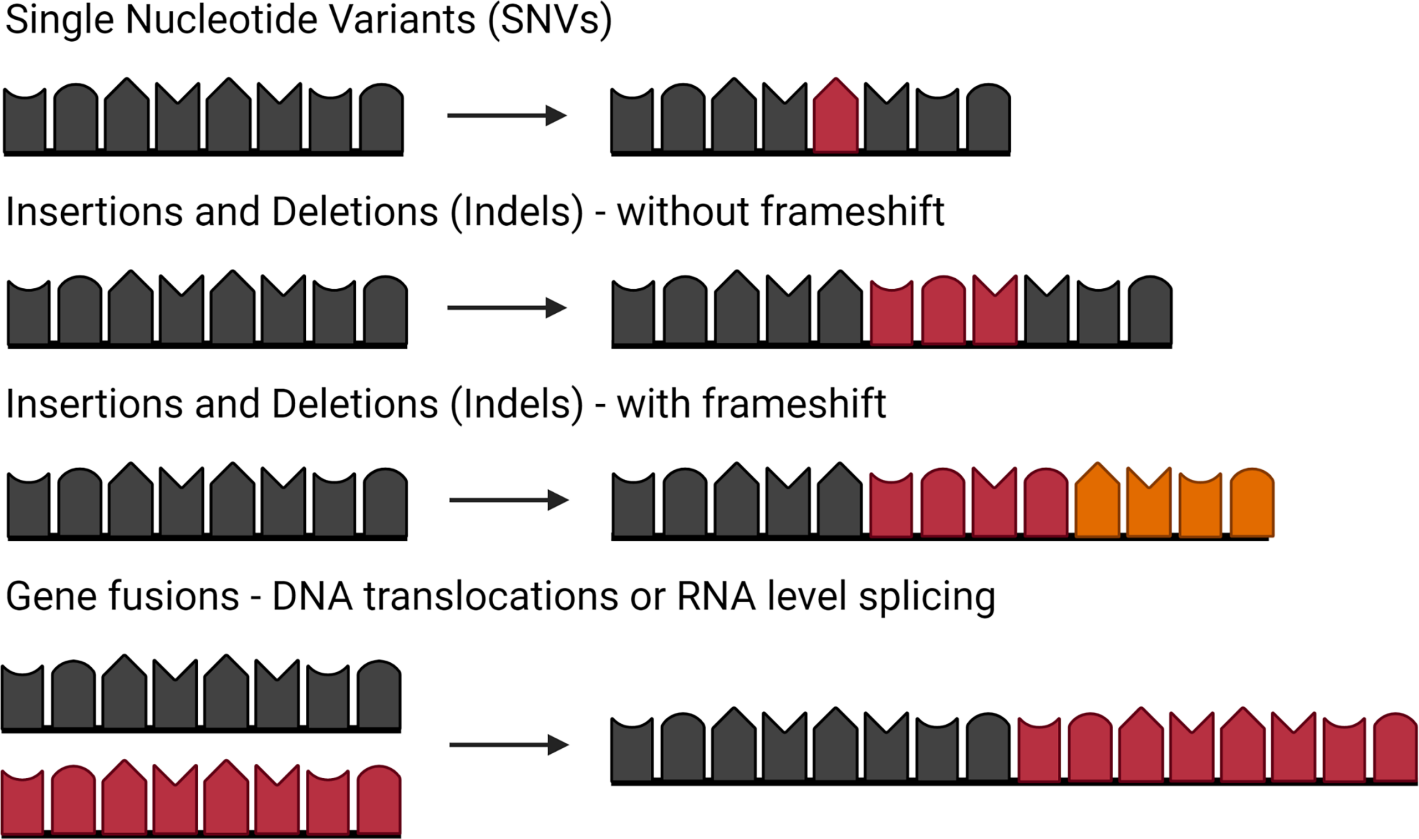
Types of mutations that can lead to neoantigens. Single nucleotide variants (SNVs) caused by a point mutation in a single nucleic acid. Insertions and deletions (indels) caused by addition of nucleic acids or loss of nucleic acids. Indels with a frameshift occur when the number of nucleic acids is not a multiple of three, changing the reading frame. Gene fusions can be caused by either translocations at the DNA level or RNA splicing of independent transcripts. Figure created with BioRender.com.

While SNVs and small indels have been the only sources of neoantigens considered in most studies to date, a growing body of evidence demonstrates the need to consider a much broader set of neoantigens (52–54) (summarized in **Figure 3**). T cells specific to a single gene fusion mutation led to complete clinical response for a patient treated with immune checkpoint inhibition, even in the absence of any other immunogenic neoantigens (54). Large indels, particularly those that induce a frameshift, have a significantly enriched percentage of neoantigens predicted to bind with high affinity to MHC class I (52). Additionally, the number of indels with a frameshift was significantly associated with response to immune checkpoint inhibitors across three independent melanoma cohorts (52). Other targets of the immune response to cancer have been suggested, including peptides selectively expressed in tumors, foreign peptides in the case of viral-mediated cancers, and peptides derived from antigen presentation in the absence of transporter associated with antigen processing (TAP) (53, 55–57). However, this review focuses specifically on neoantigens derived from mutated peptides. This section will assess the literature on identifying SNVs, small indels, DNA-level structural variants (including gene fusions, large indels, and frameshifts) and RNA-level gene fusions.

Software for identifying SNVs continue to offer very disparate reports of the mutational profile, despite being the most common variant to be identified (22, 23, 58). For example, recent work across five patients compared the SNV and indel results from Strelka2, VarScan2, and GATK Mutect2 and demonstrated that an average of 84.41% (range 77.48-92.23%) of mutations were identified by only one of the three callers, while 13.75% (range 7.21-22.17%) of mutations were identified by two of the three, and 1.83% (0.35-5.30%) by all three (23). Because of these disparities, selection of an SNV caller is a critical component of neoantigen prediction.

Despite a large number of confounding variables, a few software for identifying SNVs and small indels stand out across multiple benchmarking studies (**Tables 1**, **2**). Several confounding factors were shown to influence the performance of variant callers, including the type of validation sets employed (63), tumor purity (61), read depth of the sequencing data (59), and upstream features of the bioinformatic pipeline, such as read mapping software (62). Additionally, across the six studies summarized in **Table 1** (59–64), only nine of the 21 SNV software were tested in more than one study. Even with these confounding factors, GATK Mutect2 and Deep Variant were routinely rated as the top or second to top programs in terms of their sensitivity and specificity for detecting SNVs and small indels. When tested at different tumor purities, all variant callers demonstrated decreased performance with decreased tumor purity (61). However, TNscope and GATK Mutect2 maintained high performance for significantly lower tumor purities than the other variant callers. Deep Variant was not evaluated in this study. Overall, GATK Mutect2 and Deep Variant showed consistently high performance across multiple benchmarking studies, with GATK Mutect2 showing high performance even at lower tumor purities.

**Table 1:** Comparison of the ranking of single nucleotide variant (SNV) callers across six benchmarking studies that have been released since 2017.

Software	Supernat et al., 2018 (59)^1^	Bian et al., 2018 (60)	Pei et al., 2020 (61)	Kumaran et al., 2019 (62)	Wang et al., 2020 (63)^4^	Wang et al., 2020 (63)^5^	Hofmann et al., 2017 (64)
**Deep Variant**	#1			#1			
**GATK MuTect2**	#1	#1	#2^2^		#2	#3^7^	
**SpeedSeq**	#1						
**TNscopeS^4^**			#1^2^				
**MuSE**		#2			#1	#2	
**Strelka**					#1	#1	
**LoFreq**					#1	#2	
**JointSNVMix2**							#1
**SAMtools**				#2			#4
**MuTect**		#3			#2	#2	
**DeepSNV**							#2
**NeuSomatic**			#3^2^				
**SomaticSniper**					#3		#3
**GATK UnifiedGenotyper**							#3
**GATK Halotype Caller**				#3			#4
**VarDict**		#4			#3	#4^8^	
**VarScan**					#3		
**FreeBayes**		#4					
**VarScan2**			#4^3^				#4
**Strelka2**			#4^3^				
**TNseq^6^**			#4				

Benchmarking papers from before 2017 were excluded as they typically compared outdated software versions or compared software that are no longer maintained. Numbers and colors indicate the relative ranking based on the individual paper, with one (green) being the highest two (yellow), three (orange), and four (red) being the lowest.

^1^These rankings are based on 30x data. In 15x data, the improved performance of DeepVariant was enhanced.

^2^At 20% purity.

^3^Good performance at high purity, but poor performance for low purity samples.

^4^Results based on DREAM WGS datasets as ground truth.

^5^Results based on WES and deep sequencing spike in studies.

^6^Software not free.

^7^High performance at low VAF, low performance at high VAF.

^8^High sensitivity, but with very high false positive rate.

**Table 2:** Comparison of the ranking of insertion and deletion (indel) callers across four benchmarking studies that have been released since 2017.

Software	Supernat et al., 2018 (59)	Pei et al., 2020^1^ (61)	Kumaran et al., 2019 (62)	Wang et al., 2020 (63)
**Deep Variant**	#1		#1	
**GATK MuTect2**	#2	#1		#1
**LoFreq**				#1
**Strelka**				#1
**TNscope**		#2		
**GATK Haplotype Caller**			#2	
**VarDict**				#2
**VarScan**				#2
**SpeedSeq**	#3			
**TNseq**		#3		
**VarScan2**		#3		
**Strelka2**		#3		
**NeuSomatic**		#3		
**SAMtools**			#3	

Benchmarking papers from before 2017 were excluded as they typically compared outdated software versions or compared software that are no longer maintained. Numbers and colors indicate the relative ranking based on the individual paper, with one (green) being the highest, two (yellow), and three (orange) being the lowest.

^1^40% purity.

Consensus approaches have also been suggested, but highlight the need to balance sensitivity and specificity, especially for potential clinical applications. Wang et al. demonstrated that a majority voting approach with LoFreq, Mutect2, Strelka, and VarDict demonstrated an improved balance of precision (false discovery rate) and recall (true positive discovery rate) compared to any of the individual methods (63). Wang et al. further enhanced these results by giving increased voting power to Strelka and MuTect2 for variants with low variant allele frequency, as these variant callers demonstrated stronger performance for low frequency variants (63). For indels, results were also improved with the majority voting approach, but showed even greater improvement if a greater number of software identified the indel, suggesting higher rates of false positives among indels (63). Bian et al. demonstrated improved results, as measured by the average of sensitivity and specificity, for SNVs using a majority voting approach between FreeBayes, VarDict, and Mutect compared to individual programs (60). FreeBayes, VarDict, and Mutect were selected because they could be run with an integrated Python package, but these programs were the three callers with the worst balance of sensitivity and specificity when run individually (60). Consensus approaches present a trade-off, as they often improve specificity while decreasing sensitivity. While increasing the specificity is important to avoid testing a large number of false positive variants, lowered sensitivity increases the risk of missing a clinically important variant. Therefore, an important area for future research is to compare different combination approaches and their influence on downstream neoantigen prioritization.

Structural variants, defined as genomic alterations encompassing at least 50 base pairs, can be identified well by a single, high-quality software, and do not demonstrate a benefit from a consensus approach (65). Structural variant types include large indels (with or without a frameshift) and gene fusions (66). Several software packages have been created for the identification of structural variants. GRIDSS and Manta perform consistently well across samples, as shown by Cameron et al. in a benchmarking study evaluating precision and recall (65). An advantage of Manta is that it works well with WES or WGS, whereas GRIDSS is only applicable to WGS (67). Cameron et al. also points out the risks of a combination approach with respect to structural variants: a simple union approach can drive up the false positives significantly, whereas conservative combinations, such as intersections of two software, can lead to extremely low sensitivity. No combination approach was able to consistently outperform the results from Manta or GRIDSS independently (65). Therefore, for structural variants, the current recommendation is to employ a single, highly rated caller such as Manta or GRIDSS.

A complementary RNAseq approach to detecting gene fusions allows for both confirmation of DNA-level structural variants and the identification of RNA-level splicing events. A 2019 benchmarking study recommended the use of STAR_Fusion, Arriba, or STAR-SEQR for the identification of gene fusions from RNAseq, due to their combination of fast speed and high accuracy, as measured by the AUC (68). At this time, there have not been reported studies of benefits from combining gene fusion prediction results.

While frameshift mutations are traditionally accounted for using structural variant software, an alternative approach that may allow for identification of an expanded set of open reading frames is the use of Ribo-seq data. Ribo-seq identifies the triplet shifts of actively translating ribosomes, which allows the reading frame to be identified for all proteins being translated at the time of cell lysis (50). Ribo-seq has been proposed as a novel approach for detecting neoantigens derived from open reading frames by providing a snapshot of all active translation (51). An advantage to Ribo-seq data is that it may be able to identify novel open reading frames caused by translational dysregulation rather than by frameshifts. Further evaluation of the neoantigens identified by Ribo-seq compared to other sequencing technologies may clarify the implications of Ribo-seq technology to the clinical setting.

### 2.4 Variant Annotation

Annotating the effects of a variant on the resulting peptide sequence has high accuracy for SNVs and small indels, but accuracy drops for more complex variants such as splicing variants. Nucleotide mutations can have many potential impacts on the amino acid sequences including silent variants, variants in a non-coding region, missense mutations, frameshifts, and stop codon gain or loss. Each of these results in a significantly different set of neoantigen predictions, and therefore, variant annotation is essential to determine the neoantigen profile. Between the two most common variant annotation software, the Variant Effects Predictor (VEP) (69) and ANNOVAR (70), there was an 86.5% exact match rate overall, dropping to a low of 57.27% for splicing variants (71). Because of the difficulty in determining a “correct answer” for each variant, it is very difficult to benchmark the success of different programs. Nonetheless, based on a 2014 benchmarking study, VEP more consistently aligned with the best available, manually curated results (71). Since this benchmarking was performed before the most recent versions of either software, the results may be different with a repeated benchmarking analysis. The most recently released software, ShAn and Nirvana, have demonstrated an increase in speed and online accessibility compared to VEP with the same level of predictive abilities (72, 73). Therefore, the best available software by current recommendations is VEP for command-line applications and ShAn or Nirvana for online applications.

## 3 Neoantigen Prioritization

### 3.1 MHC Class I-Restricted Neoantigen Characteristics

**Figure 4:**
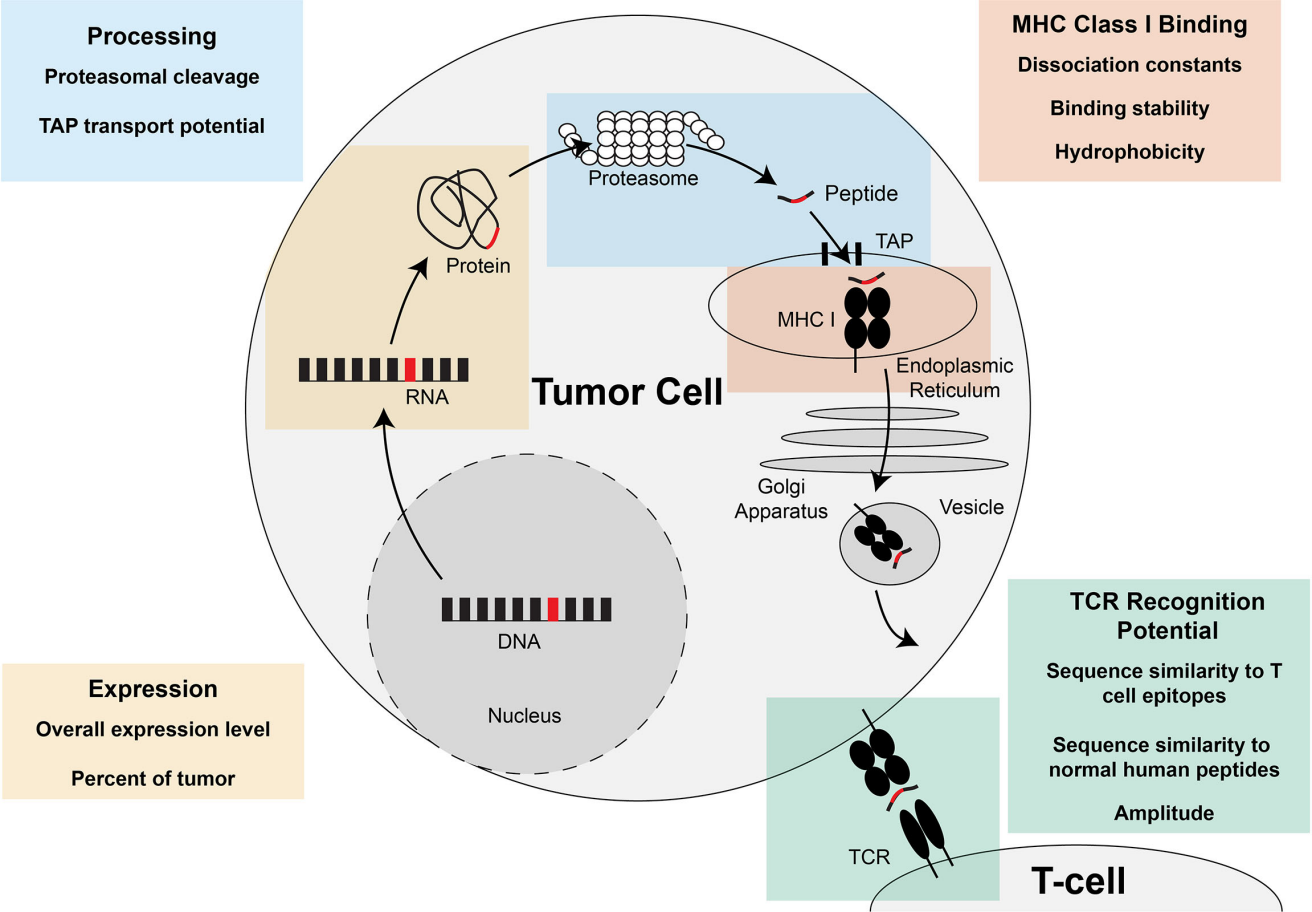
Steps of MHC class I-restricted neoantigen prioritization and summary of characteristics considered for each step. Mutations in the DNA of a tumor cell are transcribed into RNA and translated into a protein. At the end of the life cycle of the protein, the protein is broken down into peptides by the proteasome and transported into the endoplasmic reticulum by the transporter associated with antigen presentation (TAP). Once inside the endoplasmic reticulum, the peptide has the opportunity to be loaded on MHC class I. If the peptide is successfully bound to MHC class I, the peptide:MHC complex is transported to the cell surface where the peptide:MHC complex has the opportunity to be recognized by the T cell receptor (TCR). Characteristics of the neoantigen encompassing expression, processing, MHC class I binding, and TCR recognition potential have been assessed to enhance prioritization of MHC class I-restricted neoantigens and are summarized in each of the boxes in the figure.

Once the neoantigens are predicted, each neoantigen can be prioritized for therapeutic use by predicting their potential to elicit a CD8+ T cell response. The experimentally validated potential for MHC class I and II-restricted neoantigens to elicit a CD8+ or CD4+ T cell response, respectively, will be referred to as the “immunogenicity” of the neoantigen. One driving hypothesis in prioritization of immunogenic neoantigens is that the ability to predict the potential of the neoantigen to undergo each requisite step in the antigen presentation pathway will lead to improved prediction of the neoantigen immunogenicity. Tools have therefore been created to predict the expression of the neoantigen, the percentage of the tumor that contains the neoantigen of interest, the proteasomal cleavage potential, the potential for transport in the endoplasmic reticulum *via* TAP, the potential to bind the MHC class I molecule, the stability of the neoantigen:MHC class I interaction, and the potential to be recognized by a TCR (Summarized in **Figure 4**). Another body of work has focused on how to best summarize these individual tools into overall predictive models for CD8+ T cell response. Here, we will summarize the tools available for predicting each characteristic individually and then the different models available for integrating the characteristics into an overall score of the neoantigen immunogenicity.

#### 3.1.1 Expression

A neoantigen needs to be expressed within the cell in order to elicit an immune response, but the best technology to assess the expression of the neoantigen is an ongoing question. Options for assessing expression can be broken down broadly into mRNA expression, protein level expression, or active translation. mRNA expression can be assessed through RNAseq, targeted sequencing, or microarray data. RNAseq data has the advantages that it is a readily available sequencing technique and can serve as a multi-purpose dataset, contributing to variant calling and neoantigen prioritization. One limitation in the use of mRNA expression data has been isolating only the expression of the specific allele in which the variant occurs. Identification of the specific variant allele is important as there are demonstrated cases where transcription of either the mutant allele or wildtype allele is favored (74). Novel methods of selecting the allele-specific expression have been published but have not yet been applied to the field of neoantigen prioritization (74). A second limitation to the RNAseq approach is that translational regulation may lead to discrepancies between the mRNA expression in the cell and the availability of the resulting peptide to be presented by MHC. Protein level expression can be assessed through various array-based methods, as well as mass spectrometry. One method growing in popularity is complete proteomic analysis, wherein a cell is lysed, and the full protein profile of the cell is analyzed with mass spectrometry (75). The advantage of a proteomics-based approach is that it validates the presence of the mutation on the protein level, eliminating variants that may never be translated within the cell. Some limitations to the use of mass spectrometry include low sensitivity for mutated peptides and high false positives in peptide identification algorithms (75). The rapid improvement in these methods may soon ameliorate these concerns (76), but a remaining limitation is that important neoantigens may come from translational products that are rapidly degraded and would not be detected by proteomic techniques (57). Therefore, another option is the analysis of active translation occurring within a cell through the newer Ribo-seq technology. Ribo-seq data allows for quantification of all transcripts being actively translated at the time of cell lysis. Advantages of the use of Ribo-seq data are that it eliminates consideration of variants that are not translated by the cell, but also will detect translational products that are too rapidly degraded to be detected by traditional proteomic approaches (57). An important direction for future research is the comparison of RNA level, protein level, and translational level data on quantifying expression and their impact on neoantigen prioritization.

Following overall expression level, the next characteristic to consider in prioritizing immunogenicity is the percentage of the tumor that contains the variant of interest - also termed the clonality of the variant. Clonality is thought to be of particular importance for cancer therapeutics, since a variant expressed by a small, sub-clonal population of the tumor is a less attractive candidate for tumor therapy. There are a few possible ways by which to approach estimating the clonality of the variant. The ideal approach would be to use a clonal deconvolution software and then assign each neoantigen a value based on the percentage of the tumor that contains that neoantigen. Until recently, PyClone was the software most widely used (77). Recently, a newer model called FastClone was released, which demonstrated enhanced performance compared to PyClone (78). While clonal deconvolution is ideal, the programs do not always converge on a solution, especially depending on the purity and read depth of the samples. An alternative approach is to use the variant allele frequency (VAF) as a proxy for the clonality of a neoantigen, although the VAF does not account for the copy number variation, germline tissue contamination, or sample purity. Overall, as estimating the clonality of a variant is a rapidly evolving field, it is likely that enhanced deconvolution methods will continue to develop and improve.

A unique system for applying the clonality has been put forward called the CSiN score, which is applied across both MHC class I- and II-restricted neoantigens (79). The CSiN score is calculated by first calculating the product of the variant allele frequency (VAF) of each somatic mutation and the number of neoantigens that can be generated from that mutation. The overall score for the tumor is then calculated by taking the average across all mutations, weighted by the binding affinity of the neoantigens. The CSiN score is associated with survival in response to immune checkpoint inhibitors, suggesting that clonality may play a significant role in determining the potential of MHC class I and II-restricted neoantigens to elicit immune-mediated tumor destruction (79).

#### 3.1.2 Processing

One of the first steps in MHC class I-restricted antigen processing is proteasomal cleavage of proteins in the cytoplasm; incorporation of the enzyme specificity of the proteasome may lead to enhanced neoantigen prioritization. The first available model for predicting proteasomal C-terminal cleavage was NetChop (80), the method incorporated in the popular NetCTLpan model for predicting the processing and MHC binding of neoantigens. NetChop enhances the specificity of binding predictions (81). A newer model, the Proteasome Cleavage Prediction Server (PCPS), demonstrated enhanced sensitivity (0.89 vs. 0.79), but diminished specificity (0.55 vs. 0.60), compared to NetChop for discriminating known CD8+ T cell epitopes from random peptides (82). While these results are not sufficient to recommend proteasomal cleavage as an independent metric for immunogenicity, they indicate that proteasomal C-terminal cleavage may play a role in determining the neoantigen profile.

Once small peptides are generated through proteasomal cleavage, TAP transports peptides into the endoplasmic reticulum for loading onto MHC class I; predicting the specificity of TAP for certain peptide motifs may enhance neoantigen prioritization. Prediction tools for TAP transport potential are less established. Currently, the only available program for predicting TAP specificity is that integrated into the NetCTL program (81). TAP transport potential was demonstrated by the NetCTL paper to enhance specificity for MHC class I binding predictions, but decreased sensitivity at lower specificity thresholds (81). Assessment of the association between TAP transport potential and neoantigen immunogenicity has not been directly assessed. Overall, TAP transport may prove to be a useful addition to other tools, but has not shown evidence of individual predictive value for neoantigen immunogenicity.

#### 3.1.3 MHC Class I Binding

MHC class I binding affinity is one of the central neoantigen characteristics considered for prediction of neoantigen immunogenicity. Many studies have shown that MHC class I binding affinity alone has strong predictive ability for neoantigen immunogenicity (83–86). There is an abundance of models to predict MHC class I binding affinity that are summarized in **Table 3**. Binding affinity is defined as the inverse of the dissociation constant and models created to predict the binding affinity have been trained on either binding affinity alone, or binding affinity in combination with peptides eluted from MHC class I molecules and assessed by mass spectrometry. Since peptide elution does not give quantitative information regarding the binding affinity of the peptide, mass spectrometry data is included in these models as a categorical value that is integrated with the continuous binding affinity data. Many of the top performing models assess their performance with metrics such as the AUC, which measure the success of their ability to classify neoantigens as binders compared to non-binders. However, the top performing models based on AUC underperform when assessed with correlation coefficients between true and predicted binding affinities (100). As noted in **Table 3**, many models self-reported relative performance compared to other available models. In addition to the self-reported performance, three benchmarking studies have been published since 2012 which report the relative performance of the available tools. The first study found that no tool emerged as the best across all HLA alleles and all peptide lengths, but generally, artificial neural network tools outperformed those trained with other models (101). A second benchmarking study found that MHCflurry, NN_align, and NetMHCpan4.0 performed best for binding/non-binding classification. When tested specifically on mass spectrometry data, NetMHCpan4.0 and MixMHCpred show enhanced predictive power (100). Consistent with the first benchmarking study, all of these except MixMHCpred are artificial neural networks. The third benchmarking study assessed a large number of tools in terms of their ability to distinguish peptides that elicited a CD8+ T cell response. They found, similarly to the first two benchmarking studies, that NetMHCpan4.0 and MHCflurry outperformed other available models (102). Overall, neural network approaches including NetMHCpan4.0, MHCflurry, and NN_align consistently emerge as the top performing binding affinity models currently available.

**Table 3:** Comparison of available neoantigen: MHC class I binding prediction tools.

Software	Model Type	Data Type	Published Comparisons	Performance metrics
**NetMHCpan4.1 (** 86 **)**	Artificial neural network	Mass spectrometry eluted peptides and binding affinity measurements	Outperformed MHCflurry1.2 and MixMHCpred, outperformed NetMHC4.0 for HLA-B and -C	Immunogenicity predictions
**MHCflurry2.0 (** 87 **)**	Artificial neural network	Mass spectrometry eluted peptides and binding affinity measurements	Outperformed NetMHCpan4.0 and MixMHCpred	Binding vs. non-binding predictions
**MHCnuggets (** 88 **)**	Artificial neural network	Binding affinity measurements	Comparable performance to MHCflurry1.2 and NetMHCpan3.0	Binding vs. non-binding predictions
**NNAlign (** 85 **)**	Artificial neural network	Mass spectrometry eluted peptides and binding affinity measurements	Comparable performance to NetMHCpan4.0, outperformed MHCflurry1.2 and MixMHCpred	Binding vs. non-binding predictions
**ForestMHC (** 89 **)**	Artificial neural network	Mass spectrometry eluted peptides	Outperformed original NetMHC, original NetMHCpan, and MixMHCpred	Binding vs. non-binding predictions
**ACME (** 90 **)**	Artificial neural network	Binding affinity measurements	Outperformed NetMHCpan4.0	Correlation with validated binding affinity
**NetMHC4.0 (** 84 **)**	Artificial neural network	Binding affinity measurements	None provided	None provided
**MixMHCpred (** 91 **)**	Matrix approach	Mass spectrometry eluted peptides	Outperformed NetMHC3.0 and NetMHCpan3.0	Binding vs. non-binding predictions
**MSIntrinsic (** 92 **)**	Artificial neural network	Mass spectrometry eluted peptides	Outperformed NetMHC4.0 and NetMHCpan2.8	Binding vs. non-binding predictions
**ConvMHC (** 93 **)**	Artificial neural network	Binding affinity measurements	Outperformed Pickpocket, IEDB SMM, and original NetMHCpan model	Binding vs. non-binding predictions
**PAComplex (** 83 **)**	Binding models	Database of known binding peptides	None provided	None provided
**IEDB SMMPMBEC (** 94 **)**	Matrix approach	Binding affinity measurements	Outperformed IEDB SMM, underperformed original NetMHC model	Binding vs. non-binding predictions
**PickPocket1.1 (** 95 **)**	Matrix approach	Binding affinity measurements	Underperformed original NetMHCpan model	Binding vs. non-binding predictions and correlation with validated binding affinity
**IEDB recommended (** 96 **)**	Positional scanning peptide libraries	Binding affinity measurements	Only compared to older models not included in this summary, performed better than 10/16 available methods	Binding vs. non-binding predictions
**ARB (** 97 **)**	Matrix approach	Binding affinity measurements	None provided	None provided
**IEDB SMM (** 98 **)**	Matrix approach	Binding affinity measurements	None provided	None provided
**SYFPEITHI (** 99 **)**	Binding motifs	Binding affinity measurements	None provided	None provided

Models included that met the following criteria 1) released since 2012 or included in a benchmarking study since 2012, 2) published in a peer reviewed journal, 3) available for web-based or command-line application, and 4) the most recent versions of a given software. Published comparisons are based on the comparisons reported in the publication of the new model. Performance metrics summarize whether the published comparisons were based on the ability of the model to predict immunogenicity, categorize each neoantigen as a binder vs. non-binder, or on the correlation between the predicted and experimentally validated binding affinity.

A few studies have also suggested consensus approaches to the prediction of MHC class I binding, though none are currently optimized for application. For example, MHCcombine is a web application which runs 13 prediction algorithms and provides the outcome from each (100). Given that no model consistently outperformed across all peptide lengths and HLA alleles, MHCcombine may allow the user to apply the best result for the particular peptide length and HLA allele being tested. Additional research is needed on how to scale this approach for application to large lists of peptides and how these results would impact the overall performance. Another study averaged the results from early versions of NetMHCpan and NetMHC and showed a small performance enhancement (103). However, as these results predate many of the high-performing software summarized in **Table 3**, further research is needed to see how combined methods may impact performance.

Another characteristic of MHC class I binding that has been less studied is the binding stability, which is not directly assessed in any of the tools summarized above. While the binding affinity and binding stability are mathematically related, they may provide complementary information. Whereas the binding affinity, which is assessed by most available tools, is the inverse of the concentration at which 50% of the MHC class I molecules will be bound to the neoantigen, the binding stability is the half-life of the binding interaction. The binding affinity is the best metric for reactions in which the interaction of the two molecules is instantaneous. But, for the prediction of neoantigens, which must stay bound until a circulating T cell is able to recognize them, the stability of the interaction may also be important. Therefore, tools predicting the binding affinity and binding stability have been proposed to be synergistic in predicting the potential for a neoantigen to be meaningfully presented on an MHC class I molecule. There is only one program that predicts MHC class I:peptide binding stability, NetMHCstabpan (104). As noted by the creators of NetMHCstabpan, the creation of a binding stability model was limited by the relative lack of training data for stability compared to binding affinity. Despite the limited training data, recent work has demonstrated enhanced neoantigen prioritization by combining both binding affinity and binding stability predictions (22, 23). Prediction of binding stability is an area where future work may lead to substantial improvements.

The hydrophobicity of a neoantigen is an additional characteristic with the potential to impact MHC binding and TCR recognition, but has demonstrated inconsistent predictive value for neoantigen immunogenicity. Since the binding cleft of the MHC class I molecule and the CD8+ TCR contact residues are both hydrophobic, one hypothesis is that a more hydrophobic neoantigen would be more likely to bind the MHC binding cleft and TCR (105). Two independent neural network approaches demonstrated a significant association of increased hydrophobicity with increased neoantigen immunogenicity (21, 105). In contrast, the TESLA consortium calculated a hydrophobicity fraction as the number of hydrophobic neoantigens divided by the length of the neoantigen and found a significantly higher hydrophobicity fraction among non-immunogenic neoantigens (22). When the hydrophobicity fraction was applied across four independent datasets, no consistent association of hydrophobicity with immunogenicity was observed (23). The differences in the observed associations of hydrophobicity with immunogenicity may be due to differences in the hydrophobicity of different HLA alleles. Published binding motifs for peptides known to bind different HLA alleles have demonstrated dramatic differences in the conserved amino acids. For example, HLA-A02:01 has several conserved hydrophobic amino acids, whereas HLA-A01:01 has predominantly polar and charged conserved amino acids (91). The neural network models from Chowell et al. and Zhou et al. were trained on known T cell epitopes from the immune epitope database (IEDB) (106), which has an HLA-A2 allelic bias since HLA-A2 is the most common class I allele, particularly in Caucasian populations (107). HLA-A2 also has more available experimental tools, which has expanded the bias towards this allele. A similar HLA-A2 allelic bias was observed in the TESLA dataset, with HLA-A2 alleles comprising 39.3% of the data, but there was also a high percentage of several alleles known to have conserved amino acid residues that are polar or charged, including HLA-A01:01 (22). Additional research is needed to fully understand the association of hydrophobicity with immunogenicity in the context of a diverse set of HLA alleles.

For all considerations of MHC class I binding, an understanding of the HLA alleles present in the tumor is critical. Predictions of dissociation constants and stability rely on the specific HLA allele to which the neoantigen is binding. Additionally, as discussed above, there is evidence that the impact of hydrophobicity may be allele specific. Beyond the facilitation of binding and hydrophobicity predictions, changes in the HLA alleles such as mutations or loss of heterozygosity are a known mechanism of immune evasion in cancers (108). In addition, intact antigen processing machinery is required for presentation of the neoantigen and subsequent destruction by CD8+ T cells (109). Loss of functional components of the MHC class I antigen processing pathway including beta-2-microglobulin (110), TAP (111, 112), and tapasin (113, 114) have been implicated in immune-evasion or resistance to immunotherapy. Therefore, the HLA allelic profile of the tumor and the status of the antigen presentation pathways are critical to understanding which neoantigens can be presented to facilitate immune-mediated tumor destruction.

#### 3.1.4 T Cell Receptor Recognition

Another characteristic of neoantigens that has been considered for impact on neoantigen immunogenicity is the TCR recognition potential. As T cells develop in the thymus, they are exposed to self peptides. T cells that recognize self peptides with high avidity undergo apoptosis. Therefore, T cell recognition has been broadly evaluated as either the similarity of the neoantigen to a normal human peptide or the similarity of neoantigens to known T cell epitopes.

The first method, similarity of the neoantigen to a normal human peptide has been shown to decrease the likelihood of the neoantigen eliciting an immune response. Increased sequence similarity was demonstrated to be highly associated with a decreased chance of eliciting an immune response across a large set of peptides known to elicit a T cell response from the IEDB (106). Sequence similarity alone was able to predict immunogenicity with an AUC of 0.85 (115). Importantly, these peptides derive from a variety of diseases including viruses, bacteria, and cancer neoantigens. In subsequent studies restricted to tumor neoantigens, the sequence similarity has not shown a significant association with neoantigen immunogenicity (23, 26). The observed differences may be explained by the much smaller range of sequence similarity available in the tumor neoantigens tested for immunogenicity. Since most tumor-derived neoantigens that have been tested for immunogenicity derive from SNVs (discussed below), they differ by a single amino acid. By contrast, peptides from viruses could be 100% distinct from normal human peptides. Further research is needed to determine if sequence similarity is more important in predicting neoantigen immunogenicity of tumor neoantigens when a broader set of neoantigens is considered.

Another method for accounting for TCR recognition is a model developed by Łuksza et al., which integrates three neoantigen characteristics into an aggregate fitness score for the tumor and demonstrated significant association of a lower fitness score with improved response to immune checkpoint inhibition. The overall fitness score is defined by the product of the T cell recognition probability, anchor residue hydrophobicity, amplitude, and a factor of negative one. A higher value for T cell recognition, amplitude, or hydrophobicity all contribute to a lower fitness score (more negative value) and a neoantigen that is more likely to be visible to the immune system. The first characteristic, the T cell recognition potential, applies a probabilistic model for the binding of the neoantigen to the TCR by using the sequence similarity between the neoantigen and the closest matched T cell epitope from the IEDB (19, 106). The second characteristic accounts for the hydrophobicity of the neoantigen by giving the neoantigen a hydrophobicity of zero if an anchor residue is mutated from a hydrophobic residue to a hydrophilic residue, and all other changes are given a score of one. The third characteristic is called the “amplitude” and is intended to adjust for self-recognition. The amplitude is calculated as the ratio of the dissociation constant for the wildtype peptide and the neoantigen (19). The amplitude is higher for neoantigens that have a lower dissociation constant (higher binding affinity) and are derived from a wildtype peptide with a high dissociation constant. Neoantigens derived from a wildtype peptide with a high dissociation constant are predicted to be less likely to be subject to immune tolerance, since the wildtype peptide is less likely to be presented to developing T cells in the thymus. The integrated Łuksza model demonstrated a significant association of lower tumor fitness score with improved survival in patients treated with immunotherapy but was not assessed as a predictive measure for the immunogenicity of individual neoantigens (19).

Capietto et al. independently assessed the amplitude characteristic and suggested that the amplitude may be of greatest importance in predicting neoantigen immunogenicity for mutations in anchor residues (116). Capietto et al. found that the amplitude was a better predictor of immunogenicity for neoantigens with a mutation in the anchor residue than was the dissociation constant alone (116). These results suggest that the difference based on mutation position may be due to a greater change in T cell recognition when the mutation is in a non-anchor residue. However, the unadjusted binding affinity was significantly associated with immunogenicity in neoantigens with mutations in either anchor or non-anchor residues in this study and an independent study (23, 116). Further research will be needed to isolate the role of mutation position on immunogenicity predictions.

#### 3.1.5 Integrated Models

Given the large number of neoantigen characteristics and tools to consider in prioritizing immunogenicity, several papers have focused on integrating neoantigen characteristics into an overall immunogenicity score. **Table 4** summarizes six recent models based on the characteristics they include and their reported performance as an AUC when provided. Of interest, the one commonality among all models is the inclusion of the binding affinity calculated by NetMHCpan (21–26). The consistent inclusion of MHC binding affinity across all available studies highlights the importance of MHC class I binding in determining the immunogenicity of at least a subset of neoantigens. Three models (TESLA, NeoScore and Neopepsee) focused specifically on reducing the characteristics included to only those most necessary for prioritizing neoantigens (22, 23, 25). TESLA and NeoScore were trained on the same training dataset and selected the same three characteristics, with the difference being that TESLA provides a series of thresholds across the three characteristics, while NeoScore provides a continuous score. The three selected characteristics were MHC class I binding affinity, MHC class I binding stability, and mRNA expression level (22, 23). In contrast, Neopepsee selected hydrophobicity, polarity, T cell recognition potential, amplitude, and the amino acid contact potentials (25). The striking difference in the selected characteristics may reflect a difference in the underlying training datasets. Neopepsee was trained on a set of known T cell epitopes from across diseases compared to common human variants presumed to not be immunogenic, whereas the other models were trained on tumor-specific neoantigens (22, 23, 25). The final Neopepsee score was demonstrated to be associated with immunogenicity in a test set derived exclusively from tumor mutations (25). Continued research is needed to select and validate the best set of characteristics to prioritize immunogenic neoantigens

**Table 4:** Summary of MHC class I-restricted neoantigen prioritization models.

	MuPeXI (24)	Neoepitope novelty (26)	Neopepsee (25)	pTuneos (21)	TESLA (22)	NeoScore (23)
**Expression**	RNA	–	–	RNA	RNA	RNA
**Clonality**	VAF^1^	–	–	PyClone	–	–
**Cleavage**	–	–	–	NetCTLpan	–	–
**TAP^2^**	–	–	–	NetCTLpan	–	–
**Kd^3^**	NetMHCpan	NetMHCpan	NetMHCpan	NetMHCpan	NetMHCpan	NetMHCpan
**Stability**	–	–	–	–	NetMHCstabpan	NetMHCstabpan
**Hydrophobicity**	–	–	Chowell et al.	Trained neural network	–	–
**Polarity**	–	–	Chowell et al.	–	–	–
**T cell Recognition**	–	–	Sequence similarity to known epitopes	Łuksza et al.	–	–
**Sequence Similarity**	Number of mismatches	BLOSUM62 matrix	–	BLOSUM62 matrix	–	–
**Amplitude^4^**	X	–	X	X	–	–
**Viral Similarity**	–	BLOSUM62 matrix	–	–	–	–
**Amino Acid Contact Potentials**	–	–	Saethang et al.	–	–	–
**AUC^5^ (if reported)**	AUC = 0.635 in test set	AUC = 0.66 in training set	Not reported	AUC = 0.833 with 10-fold cross-validation	Cannot be calculated	AUC = 0.845 in test set

Where applicable, the tool used for each characteristic is specified. Dashes indicate that the characteristic was not included in the model and “X” indicates that the characteristic was included in a model, but that the characteristic is a fixed quantity with no specific tools to report.

^1^VAF, variant allele frequency.

^2^TAP, transporter associated with antigen processing.

^3^Kd, dissociation constant.

^4^Amplitude, ratio of the dissociation constant of the wild type peptide and neoantigen.

^5^AUC, area under the receiver operator characteristics curve.

While the models summarized above focus on predicting the immunogenicity of individual neoantigens, a few models trained specifically on the response to immune checkpoint inhibition. These models include the model from Łuksza et al. and the CSiN model which are both summarized in prior sections. The model from Łuksza et al. and the CSiN model demonstrate significant association with the response to immune checkpoint inhibition but were not tested for their potential to discriminate between individual neoantigens and their potential to elicit an immune response (19, 79). NeoScore and pTuneos have also demonstrated a significant association with response to immune checkpoint inhibition, despite being trained on the immune response to individual neoantigens (21, 23). Additional work is needed to understand the relative predictive value and clinical utility of each integrated model.

### 3.2 MHC Class II-Restricted Neoantigen Prioritization

**Figure 5:**
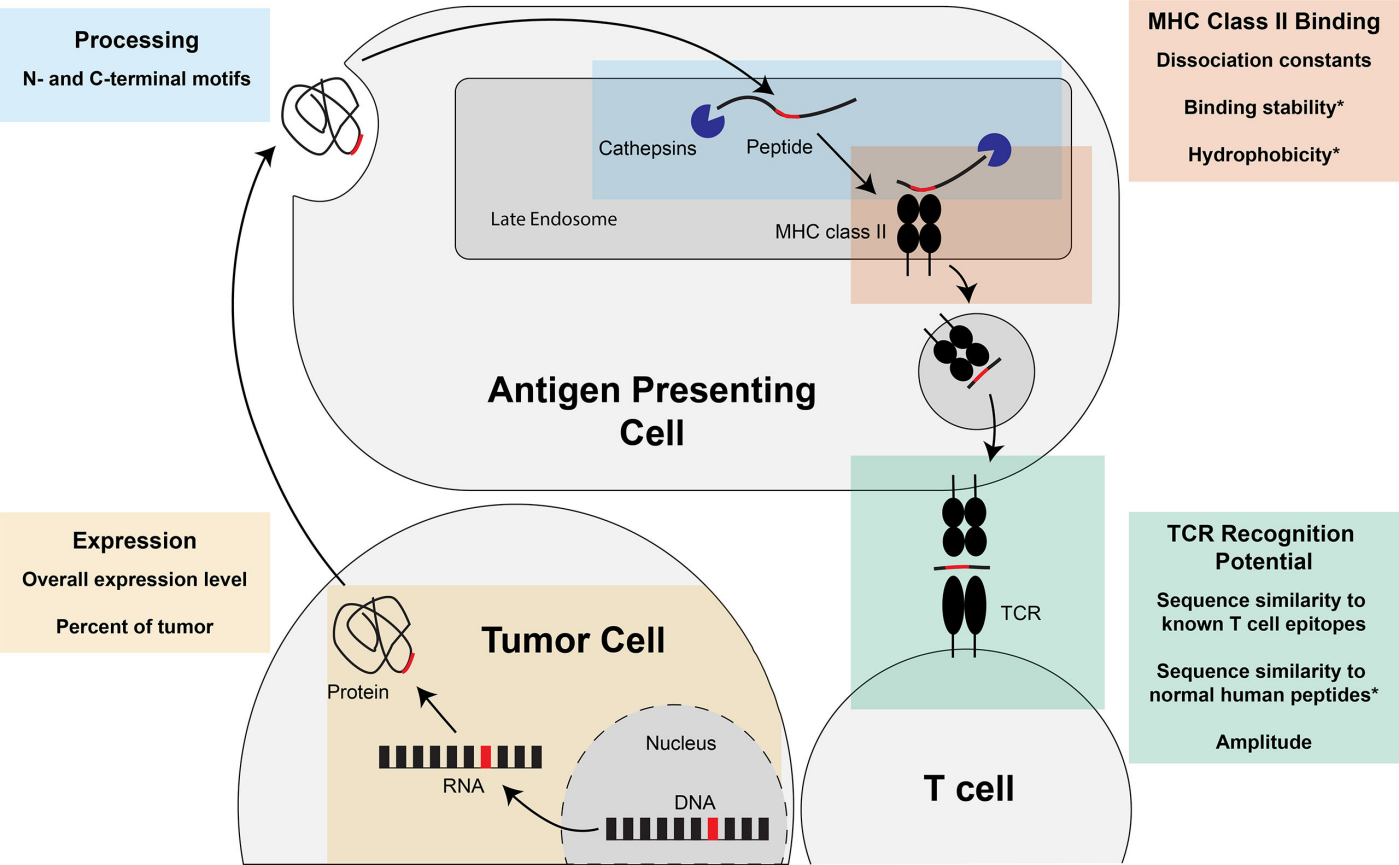
Steps of MHC class II-restricted neoantigen prioritization and summary of characteristics considered for each step. Mutations in the DNA of a tumor cell are transcribed into RNA and translated into a protein. The protein can either be taken up into the endocytic compartment of an antigen presenting cell or processed and presented by the tumor cell if the tumor cell expresses MHC class II (not pictured). In the late endosomes, protein cleavage and MHC class II loading occurs. The protein is cleaved by cathepsins at the N- and C-termini before and after binding to the MHC class II molecule. If the peptide is successfully bound to MHC class II, the peptide:MHC complex is transported to the cell surface where the peptide: MHC complex has the opportunity to be recognized by the T cell receptor (TCR). Characteristics of the neoantigen encompassing expression, processing, MHC class II binding, and TCR recognition potential that may enhance prioritization of MHC class II-restricted neoantigens are summarized in each of the boxes in the figure. * indicates characteristics that, to our knowledge, have not been assessed for the prioritization of MHC class II-restricted neoantigens.

The therapeutic applications of a neoantigen are also directly impacted by the potential of the neoantigen to bind to MHC class II and elicit a CD4+ T cell response, as CD4+ T cells have been demonstrated to play a critical role in initiating and maintaining a successful immune-mediated tumor destruction (6, 117). Prioritization of MHC class II-restricted neoantigens can incorporate many of the same characteristics as MHC class I: expression, processing, binding, and TCR recognition. Though the body of literature is smaller for prioritizing MHC class II-restricted neoantigens, tools are available to predict the expression of the neoantigen, the percentage of the tumor that contains the neoantigen of interest, the N/C-terminal cleavage potential, the potential to bind the MHC class II molecule, and the potential to be recognized by a CD4+ TCR (summarized in **Figure 5**). As for MHC class I-restricted neoantigens, there is also a body of work focused on integrating these tools into overall neoantigen immunogenicity scores. We will summarize the individual tools for each characteristic and the models available for integrating these characteristics into an overall score of the neoantigen immunogenicity.

#### 3.2.1 Expression

Expression and clonality of MHC class II-restricted neoantigens can be calculated with the same tools as for MHC class I-restricted neoantigens.

#### 3.2.2 Processing

Cleavage of peptides for MHC class II occurs in the endocytic pathway and is performed by cathepsins. The current understanding of cleavage of peptides for MHC class II is that cleavage occurs both before and after binding of the peptide to the MHC class II molecule (118). Cleavage before and after binding is supported by binding of large proteins with exposed binding motifs in the absence of any protease activity (119) and dominant binding of accessible regions of proteins over high-affinity binders that are not solvent accessible (120). Abelin et al. applied the current understanding of MHC class II-restricted neoantigen processing to create models for predicting MHC class II-restricted neoantigens. Abelin et al. assessed the solvent accessibility of different regions of the protein at the pH of the late endosome to account for binding before processing and the N- and C-terminal motifs to account for enzyme specificity of the cathepsins (121). Abelin et al. demonstrated enhanced prioritization of neoantigens that bind MHC class II based on specific N- and C-terminal motifs, but did not find an impact of solvent accessibility (121). These results are in concordance with several other models which have demonstrated an ability to improve neoantigen prioritization by identifying specific motifs (122, 123). These studies combine to suggest the importance of considering N- and C-terminal motifs in the prioritization of MHC class II-restricted neoantigens.

#### 3.2.3 MHC Class II Binding

There are many tools to predict the MHC class II binding affinity and a few that stand out as top candidates. A potentially helpful resource is the IEDB MHC II automated server benchmarks (124). The IEDB automated benchmarking system releases weekly scoring reports, ranking available MHC class II binding predictions based on the performance of the model in the most recently updated IEDB test datasets. While the IEDB automated benchmarking system has the potential to be useful for research purposes, it is currently limited by only having six software registered, all of which were published by or before 2015. A benchmarking study of a set of older tools compared to two newer tools, NetMHCIIpan3.2 and DeepSeqPanII, demonstrated a distinct jump in performance between the older and newer tools (125). Because of the large gap in performance, only a set of the five newest models is included in **Table 5**. Based on the published data, NetMHCIIpan4.0 and DeepSeqPanII are likely the best performing models currently available (125). One newer method, NeonMHC, demonstrated enhanced positive predictive value compared to NetMHCIIpan3.1 (121). However, no direct comparison has been done of NeonMHC to updated versions of the other software, suggesting that further comparison studies between these techniques may be beneficial. While MHC class II binding affinity models have improved dramatically in the last few years, side-by-side comparisons of MHC class I and II binding affinity prediction models demonstrate that MHC class II binding affinity predictions still have lower performance than binding affinity predictions for MHC class I (86). This highlights the importance of continued research and model development for MHC class II binding affinity prediction.

**Table 5:** Comparison of available neoantigen: MHC class II binding prediction tools.

Software	Model Type	Data Type	Published Comparisons	Performance Metrics
**DeepSeqPanII (** 125 **)**	Artificial neural network	Binding affinity measurements	Comparable performance to NetMHCIIpan3.2, outperformed models from before 2012	Binding vs. non-binding predictions and correlation with validated binding affinity
**NetMHCIIpan4.0 (** 86 **)**	Artificial neural network	Mass spectrometry eluted peptides and binding affinity measurements	Outperformed NetMHCIIpan3.2, MixMHC2Pred, MHCnuggets, and DeepSeqPanII	Immunogenicity predictions
**MHCnuggets (** 88 **)**	Artificial neural network	Binding affinity measurements	Comparable performance to NetMHCIIpan3.2	Binding vs. non-binding predictions
**MixMHCIIpred (** 126 **)**	Matrix approach	Mass spectrometry eluted peptides	Outperformed NetMHCIIpan3.2	Binding vs. non-binding predictions
**NeonMHC (** 121 **)**	Artificial neural network	Mass spectrometry eluted peptides	Outperformed NetMHCpan3.1	Binding vs. non-binding predictions

Five of the newest methods summarized here due to recent benchmarking demonstrating that these methods highly outperformed earlier models. Only the most recent version of each software is included. Published comparisons are based on the comparisons reported in the publication of the new model. Performance metrics summarize whether the published comparisons were based on the ability of the model to predict immunogenicity, categorize each neoantigen as a binder vs. non-binder, or on the correlation between the predicted and experimentally validated binding affinity.

There is evidence that a hydrophobicity-type approach to MHC class II binding may be worth exploring. To our knowledge a hydrophobicity model has not yet been attempted for MHC class II-restricted neoantigens. For HLA-DR, crystal structures have demonstrated that the binding cleft is hydrophobic (127). Additional, complementary evidence has demonstrated that there are two cooperative, hydrophobic binding pockets on HLA-DR which are thought to be primarily responsible for binding of MHC class II-restricted neoantigens (128). Similar to MHC class I-restricted neoantigen prediction, the hypothesis is that, given the hydrophobicity of key binding pockets in the MHC class II binding groove and the TCR contact residues, increased neoantigen hydrophobicity may lead to increased immunogenicity. Overall, hydrophobicity is a characteristic of MHC class II-restricted neoantigens that will require additional research.

#### 3.2.4 T Cell Receptor Recognition

Studies predicting the T cell recognition of MHC class II-restricted neoantigens have also been limited to date. Dhanda et al. trained neural networks using known T cell epitopes and demonstrated an AUC of 0.725 (129). This study suggests that there is potential for using known T cell epitopes to determine the probability of eliciting a T cell response, although more work is needed to enhance these predictions. An integrated model from Alspach et al. (discussed below) considered the amplitude characteristic and demonstrated that a neoantigen with a high amplitude was validated to be immunogenic (117). Additional work is needed to assess the impact of T cell recognition and immune tolerance on MHC class II-restricted neoantigen immunogenicity, whether measured by sequence similarity, amplitude, or a novel method.

#### 3.2.5 Integrated Models

A few models have integrated multiple MHC class II-restricted neoantigen characteristics into an overall predictive model for MHC class II-restricted neoantigen immunogenicity. Three of the most recent models are summarized in **Table 6**. All three models have demonstrated particularly strong performance in predicting the presentation of neoantigens on MHC class II, with Abelin et al. demonstrating the strongest predictive value (AUC = 0.98) (121). Because of the limited data available for experimentally validated CD4+ T cell responses, all three of these models were built on predicting MHC class II presentation rather than neoantigen immunogenicity. The MARIA model was subsequently tested on two available datasets for MHC class II-restricted neoantigen immunogenicity and demonstrated significant association with T cell responses when split into a high, medium, and low immunogenicity score (130). The model by Abelin et al. was used to predict top candidates for immunogenicity and 8/12 tested neoantigens elicited a CD4+ T cell response, suggesting good predictive ability for immunogenic neoantigens (121). Finally, the Alspach et al. model (trained in mouse data) was integrated with amplitude and expression data, and a CD4+ T cell response was observed for the top predicted neoantigen candidate (117). Testing of these models on expanded sets of neoantigens validated to elicit a CD4+ T cell response would be useful to further understand their performance capabilities and areas for improvement.

**Table 6:** Summary of MHC class II-restricted neoantigen prioritization models.

	MARIA (130)	Abelin (121)	Alspach (117)
**Expression**	RNA	RNA	–
**Processing**	Neural network for N-/C-terminal motifs	Neural network for N-/C-terminal motifs	–
**MHC class II Binding**	Neural network for MHC class II binding scores	NeonMHC	Hidden Markov model
		Overlap with known HLA-DQ peptides	
**Gene Bias**	–	Weighted for genes over-represented on MHC class II	–
**AUC^1^ for Predicted MHC class II Presentation**	AUC = 0.89	AUC = 0.98	AUC = 0.90

Where applicable, the tool used for each characteristic is specified. Dashes indicate that the characteristic was not included in the model.

^1^AUC stands for area under the receiver operator characteristics curve.

## 4 Neoantigen Validation

The development of prioritization models for MHC class I- and II-restricted neoantigens is reliant on the availability of datasets with validated CD8+ and CD4+ T cell responses, respectively. Generating a neoantigen validation dataset requires identification of mutations, prioritization of neoantigens to test, and testing of the neoantigens. A number of validation sets are available for MHC class I-restricted neoantigens (**Table 7**), but a far more limited selection of validation sets are available for MHC class II-restricted neoantigens (**Table 8**). The creation of a neoantigen validation set requires a number of choices regarding the mutations to be validated, the methods by which to select which neoantigens to test, and the experimental validation methods employed. This section will summarize the standard methods used for generating validation datasets to date and highlight potential areas for further research.

**Table 7:** Available sets of MHC class I-restricted neoantigens validated to elicit a CD8+ T cell response.

Author and Year	Tumor Type	Tested Neoantigens: Immunogenic Neoantigens	Available Sequencing Data	Mutations Tested	Prioritization Method	Validation Method
**Robbins et al., 2013** (131)	Melanoma	227:10	WES	SNVs and small indels	NetMHCpan2.4	ELISPOT
**Wick et al., 2014** (132)	Ovarian cancer	114:1	WES	SNVs	NetMHCpan2.4	ELISPOT
**Rajasagi et al., 2014** (133)	Chronic lymphocytic leukemia	48:3	WES	SNVs	NetMHCpan2.4	ELISPOT
**Cohen et al., 2015** (134)	Melanoma	357:9	WES, RNAseq	SNVs	Expression >1 FPKM and MHC binding by IEDB	ELISA
**Carreno et al., 2015** (5)	Melanoma	21:11	WES, RNAseq	SNVs	NetMHC2.4	ELISA
**McGranahan et al., 2016** (135)	Lung cancer	355:2	WES	SNVs	NetMHCpan2.8	Multimers
**Strønen et al., 2016** (136)	Melanoma	57:11	WES, RNAseq	SNVs	Expression >0 FPKM and NetMHC3.2, NetMHCpan2.0	Multimers
**Bentzen et al., 2016** (137)	Lung cancer	702:9	WES	SNVs	NetMHCpan2.8	Multimers
**Gros et al., 2016** (138)	Melanoma	27:6	WES, RNAseq	SNVs	VAF>10%, mutation in DNA and RNA	ELISPOT
**Ott et al., 2017** (6)	Melanoma	165:18	WES, RNAseq	SNVs and small indels	NetMHCpan2.4 and oncogene mutations	ELISPOT
**Wells et al., 2020** (22)	Melanoma and Lung cancer	347:27 (available)	WES, RNAseq	SNVs and small indels	Consensus from 25 groups	Multimers

Datasets were only included if they included a minimum of ten neoantigens.

**Table 8:** Available sets of MHC class II-restricted neoantigens validated to elicit a CD4+ T cell response.

Author and Year	Tumor Type	Neoantigens Tested : Immunogenic Neoantigens	Available Sequencing Data	Mutations Tested	Prioritization Method	Validation Method
**Ott et al., 2017** (6)	Melanoma	165:80	WES, RNAseq	SNVs and small indels	NetMHCpan2.4 and oncogene mutations	ELISPOT
**Sahin et al., 2017** (7)	Melanoma	125:60	WES, RNAseq	SNVs	RNA expression > 10 RPKM and IEDB binding predictions	ELISPOT

Datasets were only included if they included a minimum of ten neoantigens.

Neoantigen validation sets have traditionally focused on SNV and small indel-derived neoantigens, though expansion to a larger set of mutations may be an important future direction in the field. As demonstrated in **Tables 7**, **8**, all available datasets have validated SNVs and small indels (5–7, 22, 131–138). The abundance of data has allowed for the creation and testing of many models for neoantigen prioritization. However, expanding the mutations tested has the potential to illuminate if there are different characteristics that are important for neoantigens derived from a broader set of mutations. One characteristic that may be particularly impacted by expanded sets of mutations is the sequence similarity. SNVs change only a single amino acid in a protein, leaving most of the neoantigen unaltered. While indels may have slightly greater changes, these represent a minority of neoantigens validated to date. By contrast, neoantigens from novel open reading frames, gene fusions, or large indels may have over 50% of the neoantigen changed compared to the corresponding wildtype peptide. Given recent evidence of the increased immunogenicity of large indels compared to SNVs (52), the inclusion of these neoantigens may enhance the importance of the sequence similarity, which has a small range when considering only SNVs and small indels. Additionally, inclusion of an expanded set of mutations may enhance the clinical applications of available neoantigen prioritization models. Currently available MHC class I and II models are trained on mutations derived from SNVs and indels and are not trained on other mutations such as frameshifts and gene fusions (21–26, 117, 121, 130). Therefore, expanding validation sets would pave the way to allow these models to expand the neoantigens considered. A recent report demonstrated that a single gene fusion neoantigen was able to drive complete disease response in a patient (54), which further underscores the importance of considering additional sources of mutations beyond SNVs and indels as candidates for personalized cancer vaccines.

Expanding the subsets of neoantigens tested may also contribute to enhanced models for MHC class I- and II-restricted neoantigen prioritization. In order to select a reasonable number of neoantigens for validation, candidates are typically prioritized by one or more neoantigen characteristics before validation. As demonstrated in **Tables 7**, **8**, neoantigens are nearly universally prioritized by MHC binding, and in the majority of cases, by NetMHCpan predicted binding. Given the low prevalence of immunogenic neoantigens, pre-filtering is important to ensure that some immunogenic neoantigens are identified. However, the pre-filtering of neoantigens does represent a bias in the selection of optimal neoantigen characteristics. The use of the binding as a criterion is limited by the predictive power of the MHC class I binding prediction tool employed. Furthermore, work in mice has demonstrated that neoantigens with a dissociation constant experimentally validated to be orders of magnitude above 500 nM (the typical cutoff used) successfully elicited a CD8+ T cell response (139). The observation that neoantigens with low binding affinity (high dissociation constants) can elicit a CD8+ T cell response suggests that there may be additional characteristics at play in determining the neoantigen immunogenicity. However, building neoantigen prioritization models on existing datasets cannot assess these other characteristics as effectively since none of the tested neoantigens have low predicted binding affinity. While model building with the same validation datasets may enhance our ability to prioritize the neoantigens that are known to be candidates, it also has the potential to bias the field away from classes of neoantigens that have not been explored in as great of depth.

**Figure 6:**
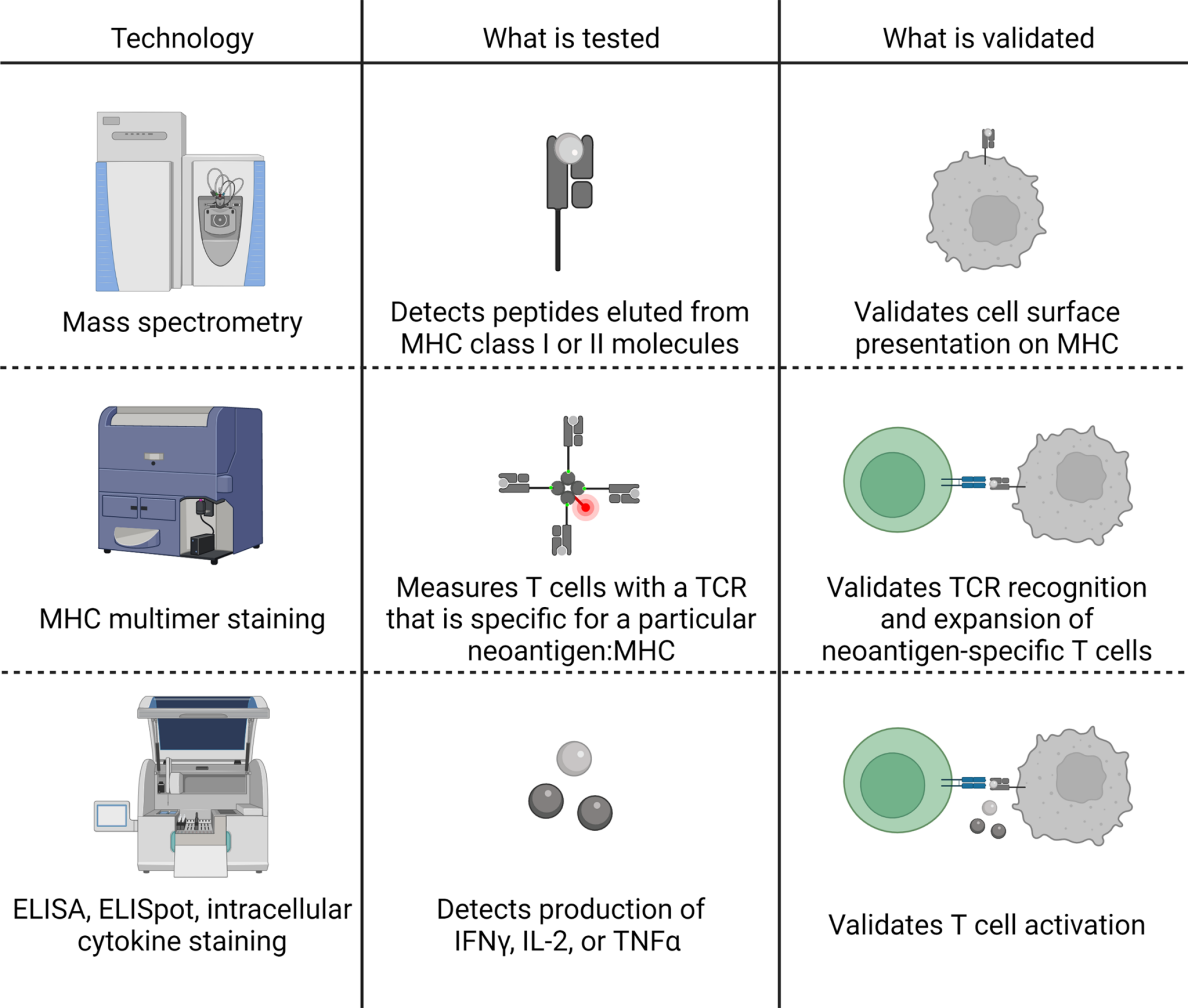
Summary of three commonly applied validation techniques for the immunogenicity of MHC class I or II-restricted neoantigens. Mass spectrometry is performed by eluting peptides directly from tumor cells and validates the *in vivo* presentation of the neoantigen on the cell surface. MHC multimers (most commonly a tetramer) bind T cell receptors (TCR) specific for the particular neoantigen: MHC, validating TCR recognition of the neoantigen and expansion of neoantigen-specific T cells. ELISA, ELISpot, and intracellular cytokine staining detect the production of cytokines, typically interferon-gamma (IFNγ), interleukin-2 (IL-2), or tumor necrosis factor alpha (TNFα), to validate T cell activation. Figure created with BioRender.com.

Validation of immunogenic neoantigens can be done in multiple ways, which all provide slightly different and complementary information. In this review, we focus on methods that involve the direct challenge of a T cell with a neoantigen. Other methods such as TCR profiling are available and have been recently reviewed (140). The standard validation techniques employed are mass spectrometry, tetramer/multimer staining, and ELISpot, ELISA, or intracellular cytokine staining, which are illustrated in **Figure 6**. These methods measure three different features of the neoantigen, and therefore, provide information about different aspects of neoantigen binding and immunogenicity. Mass spectrometry has been employed to directly profile the neoantigens presented on MHC class I and II by eluting bound peptides and identifying them using tumor-specific variant libraries (121). Mass spectrometry of eluted peptides validates MHC class I or II presentation, but must be combined with one of the other techniques to provide T cell recognition data. MHC multimers (sets of multiple MHC molecules complexed to a neoantigen of interest) bind the TCR and can be fluorescently labeled and used to stain T cells that recognize the neoantigen, a process called “multimer staining.” Multimer staining directly measures the presence of neoantigen-specific T cells that have expanded populations after activation. One feature of multimer staining that is important to keep in mind is that smaller multimers, such as tetramers, have a tendency not to stain low affinity T cells (141, 142). Given that the affinity of T cells responsive to cancer has been shown to be much lower than anti-viral neoantigens (143), using advanced methodologies for increasing sensitivity might be particularly useful in the study of tumor-specific neoantigens. The final group of techniques, ELISpot, ELISA, and intracellular cytokine staining, all test for cytokine production after stimulation of the TCR, a sign of T cell activation. One potential limitation of techniques that measure cytokine production is that these methods can give false negatives if a neoantigen-specific T cell becomes exhausted. Overall, each of the techniques provides valuable information regarding the binding or immunogenicity of the neoantigen. Where possible, combining two or more techniques may provide the best confirmation of immunogenicity.

Another consideration, both in the generation and application of neoantigen validation sets, is the differences in the neoantigen characteristics that can be validated in vaccine datasets. A common source of validated neoantigens is from vaccination studies (5–7, 136). While testing after vaccination has the advantage of demonstrating whether a given neoantigen has the potential to elicit a T cell response, it limits the validation of key, tumor-level characteristics such as expression. As recently demonstrated, models that incorporate expression underperform on these datasets (23). The underperformance of models incorporating expression is likely because the presence of a T cell response in a vaccinated patient is not necessarily due to the T cells encountering that neoantigen within the tumor. Rather, the T cells could have been activated by the vaccine, even if the tumor did not express the neoantigen of interest. Therefore, vaccination with neoantigens prior to testing presents an important consideration, both in the creation of neoantigen validation sets and in their application to validating the impact of various neoantigen characteristics on neoantigen immunogenicity.

Overall, a significant body of work has been done particularly for MHC class I-restricted neoantigen validation. Moving forward, there are several key areas that may enhance the development of clinically useful prioritization models. Specifically, these areas include 1) expansion of validation sets for MHC class II-restricted neoantigens, 2) expansion of the types of mutations considered in neoantigen validation, and 3) careful selection of which neoantigens to test for immunogenicity. Further research in these areas has the potential to build on the work already done to advance the utility of neoantigen prioritization models.

## 5 Conclusion

The field of neoantigen prediction and prioritization for cancer therapeutics has made tremendous strides and is still rapidly expanding. Prioritization of immunogenic neoantigens can be largely broken down into data acquisition and variant calling, neoantigen prioritization, and neoantigen validation. High quality sequencing data is becoming ever more accessible, and techniques for artifact removal in FFPE data and tumor-only variant calling are rapidly expanding, increasing what is feasible in each of these areas. One of the central questions in variant calling is how to find the appropriate balance between sensitivity and specificity for the clinical applications of neoantigens. While using consensus approaches between several variant calling software has the potential to enhance specificity, it may do so at the expense of missing clinically important variants. Within neoantigen prioritization, a wide range of high-performance tools are available for prioritizing MHC class I- and II-restricted neoantigens. However, MHC class II tools generally have not been assessed to the same degree as those for MHC class I, representing a key area for future research. Other key areas for enhancing neoantigen prioritization models include 1) training models directly on predicting the potential of an MHC class II-restricted neoantigen to elicit a CD4+ T cell response and 2) expanding models to include neoantigens derived from other sources of mutations. Advances in these areas will rely on the expansion of available neoantigen validation datasets with a specific focus on MHC class II-restricted neoantigens and neoantigens derived from large indels, gene fusions, or frameshifts. Overall, a combination of expanding datasets and continued improvement of computational modelling will build on past successes to create more clinically relevant models moving forward.

## Author Contributions

Writing – original draft preparation, EB. Writing – review and editing, KH, MW, KB, and EB. Visualization, EB. Supervision, KH, MW, and KB. Project administration, KH. Funding acquisition, KH and EB. All authors have read and agreed to the published version of the manuscript.

## Funding

This work was supported in part by the Springboard Initiative from the University of Arizona College of Medicine-Phoenix (KH), Merit Review Award I01-BX005336 from the United States Department of Veterans Affairs (VA), Biomedical Laboratory Research and Development Service (KH), the University of Arizona College of Medicine-Phoenix M.D./Ph.D. Program (EB), and the 2021 Melanoma Research Foundation Medical Student Award (EB). The contents do not represent the views of the VA or the United States Government.

## Conflict of Interest

The authors declare that the research was conducted in the absence of any commercial or financial relationships that could be construed as a potential conflict of interest.

## Publisher’s Note

All claims expressed in this article are solely those of the authors and do not necessarily represent those of their affiliated organizations, or those of the publisher, the editors and the reviewers. Any product that may be evaluated in this article, or claim that may be made by its manufacturer, is not guaranteed or endorsed by the publisher.
